# Phlebotomine sand flies and *Leishmania* species in a focus of cutaneous leishmaniasis in Algeria

**DOI:** 10.1371/journal.pntd.0008024

**Published:** 2020-02-18

**Authors:** Roumaissa Gherbi, Mustapha Bounechada, Maria Stefania Latrofa, Giada Annoscia, Viviana Domenica Tarallo, Filipe Dantas-Torres, Domenico Otranto

**Affiliations:** 1 Laboratory of Improvement and Development of Plant and Animal Production, University of Ferhat Abbas, Setif, Algeria; 2 Dipartimento di Medicina Veterinaria, Università degli Studi di Bari, Valenzano, Bari, Italy; 3 Department of Immunology, Aggeu Magalhães Institute (Fiocruz), Recife, Brazil; 4 Department of Pathobiology, Faculty of Veterinary Science, Bu-Ali Sina University, Felestin Sq., Hamedan, Iran; Faculty of Science, Ain Shams University (ASU), EGYPT

## Abstract

Cutaneous leishmaniasis is a disease caused by various *Leishmania* spp., which are transmitted by phlebotomine sand flies. Algeria is one of the most affected countries, with thousands of cutaneous leishmaniasis cases registered every year. From March to November of 2016 and 2017, sand flies were collected in 12 municipalities in Setif province, North-Eastern Algeria. Sand flies were identified and females were tested by PCR for detecting *Leishmania* DNA. Additionally, cutaneous leishmaniasis cases notified during the study period were analysed. Out of 1804 sand flies collected, 1737 were identified as belonging to seven species, with *Phlebotomus perniciosus* (76.2%), *Ph*. *papatasi* (16.7%) and *Ph*. *sergenti* (5.0%) being the most common species, representing together 97.9% of the collected specimens. The remaining specimens were identified as *Sergentomyia minuta*, *Se*. *fallax*, *Ph*. *longicuspis* and *Ph*. *perfiliewi*. The number of sand flies collected monthly was positively correlated with temperature. Out of 804 females tested, nine *Ph*. *perniciosus* (1.1%) scored positive for *Leishmania infantum* (n = 5), *L*. *major* (n = 3) and *L*. *tropica* (n = 1), respectively. During the study period, 34 cutaneous leishmaniasis cases were notified in Setif, of which 58.8% were patients residing in two urban and peri-urban municipalities and 41.2% in rural areas. The finding of *Ph*. *perniciosus* as the most abundant species in Setif suggests that this sand fly may be adapted to different biotopes in the North-East region of Algeria. The detection of different *Leishmania* spp. in *Ph*. *perniciosus* suggests a complex epidemiological picture of cutaneous leishmaniasis in Setif, with the involvement of different etiological agents and possibly with different reservoir hosts and vectors.

## Author summary

Leishmaniasis is a group of neglected diseases, with more than 350 million people at risk and 2 million new cases every year. *Leishmania* parasites are transmitted by phlebotomine sand flies. Algeria is one of most affected countries, with thousands of cutaneous leishmaniasis cases registered every year. However, in spite of the scientific knowledge gained over the last decades, the understanding of the biology and the ecology of sand flies in some areas of Algeria is still fragmentary. The present study was conducted from 2016 to 2017 to assess the sand fly population in Setif and also the *Leishmania* spp. circulating in this province. Our data suggest a complex epidemiological picture of cutaneous leishmaniasis in Setif, with the involvement of different etiological agents and possibly with different reservoir hosts and vectors.

## Introduction

Phlebotomine sand flies (Diptera, Psychodidae) are hematophagous insects involved in the transmission of viruses (Bunyaviridae, Reoviridae and Rhabdoviridae), bacteria (*Bartonella bacilliformis*) and protozoa (*Leishmania* spp.) to animals and humans [1–3]. Among protozoa, *Leishmania* spp. are recognized as pathogenic to humans, causing different clinical forms: visceral (VL), cutaneous (CL), mucocutaneous, post-kala-azar dermal and mucosal leishmaniasis [4]. Leishmaniases are neglected diseases worldwide distributed, occurring mainly in tropical and subtropical zones [5]. In Algeria, VL and CL are known to be prevalent since the beginning of the 20^th^ century [6–8]. Nowadays, Algeria represents the second largest focus of CL, after Afghanistan, with an incidence of 28.19 cases per 100,000 inhabitants in 2017 (http://www.insp.dz/images/PDF/Epidemio/REM%20annuel2017f.pdf).

The geographical distribution of CL and VL is closely associated to the sand fly population structure and distribution [9,10]. In Algeria, 24 sand fly species of the genera *Phlebotomus* and *Sergentomyia* have been identified, with *Phlebotomus perniciosus*, *Ph*. *perfiliewi*, *Ph*. *longicuspis*, *Ph*. *papatasi* and *Ph*. *sergenti* being proven or suspected vectors of *Leishmania* spp. to humans [11,12]. Nonetheless, data about the environmental factors that may affect the dispersion and population dynamics of sand flies, are still poor understood in some areas of this country. This is the case of Setif province, where 1288 cases of human CL and 501 of VL have been reported to the public health authorities from 1993 to 2017. The present study focused on: i) the evaluation of the diversity and seasonality of sand flies in rural and urban areas and ii) detection of *Leishmania* spp. DNA in sand fly of Setif province. We also analysed data on CL cases notified in Setif during the study period.

## Materials and methods

### Study area

**Fig 1:**
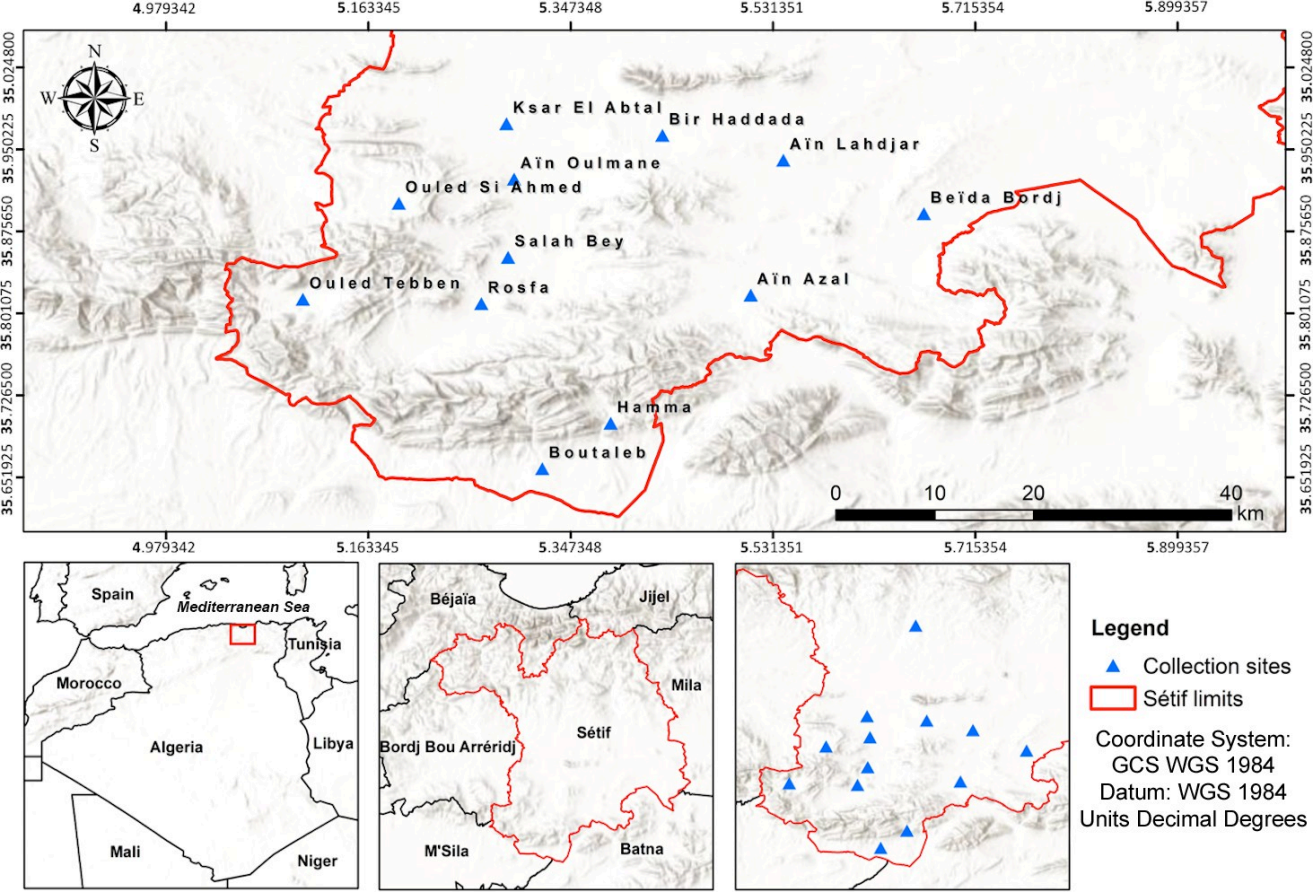
Geographic locations of the phlebotomine sand fly collection sites in Setif province, North-Eastern Algeria. The map was created using ArcMap 10.6 [19] (publicly available shapefiles from the GADM (Global Administrative Areas) DataBase (https://gadm.org/) and the ArcGIS Online basemap.

Sand flies were collected in Setif (36°11'29”N, 5°24'34”E), a semi-arid region belonging to the highland of North-Eastern Algeria (Table 1, Fig 1), with an extension of 6550 km^2^ and 1,489,979 inhabitants. This area is characterized by hot and dry summer (from the end of May until the beginning of October) with an average annual precipitation of 402 mm. The monthly average temperature reaches 5.8°C in January and 24.8°C in July, with monthly average relative humidity of 37% in July and of 79.1% in December. The monthly average speed of the wind is 2.9 m/s, with highest values (7.4 m/s) being recorded during April and May.

**Table 1:** Description of the phlebotomine sand fly collection sites in Setif province, Northeastern Algeria. The presence of animals and humans are also reported.

Municipality (ID)	Sites (ID)	Site type	Coordinates	Altitude (m)	Description ^a^
Boutaleb (A)	Lahddada (1)	Rural	35°39'49.7"N 5°16'52.1"E	826	Sheep, goat and cattle shelters
	Bni Lmai (2)		35°42'26.6"N 5°19'03.3"E	1455	Sheep and horse shelters
	Boutaleb (3)		35°41'29.1"N 5°14'51.4"E	792	Mixed shelter (cattle, sheep, poultry)
Hamma (B)	Lhammam (1)	Rural	35°43'25.1"N 5°22'06.7"E	1130	Goat shelter
	Aadaoua (2)		35°40'48.8"N 5°22'08.9"E	854	Discharge of water waste
Ouled Tebbane (C)	Lemtarih (1)	Rural	35°46'52.1"N, 5°06'25.5"E	1112	Cattle shelter
	Djbel Dahane (2)		35°47'37.4"N 5°05'21.5"E	1108	Mixed shelter (rodent, poultry, cattle, sheep)
Rasfa (D)	Rasfa (1)	Rural	35°46'47.1"N 5°14'49.4"E	1018	Sheep shelter
Ouled Sid Ahmed (E)	Djbel Osmane (1)	Rural	35°53'48.9"N 5°12'53.3"E	1259	Mixed shelters (cattle, sheep, poultry)
					Discharge of domestic waste water No human presence
Saleh Bey (F)	Chouiette (1)	Urban	35°50'01.6"N 5°16'55.6"E	976	Mixed shelter (poultry, cattle, sheep)
Ain Oulmen (G)	Ain Oulmen (1)	Urban	35°56'05.8"N 5°18'30.6"E	948	Cattle shelter
					Water treatment station
	Djbel Baadache (2)		35°54'05.5"N 5°20'44.0"E	1122	Sheep shelter
Ksar El Abtal (H)	Zmala (1)	Peri-urban	35°58'28.8"N 5°15'26.9"E	758	Mixed shelter (cattle, sheep)
Bir Haddada (I)	Bir haddada (1)	Peri-urban	35°58'09.1"N 5°29'42.6"E	930	Goat shelter
	Chouaoura (2)	Peri-urban	35°57'42.8"N 5°26'37.2"E	932	Old building
Ain Azel (J)	Ain Azel (1)	Peri-urban	35°50'07.2"N 5°31'35.6"E	925	Cattle shelter
Ain Lahdjar (K)	Ain Lahdjar (1)	Rural	36°00'01.8"N 5°33'44.5"E	936	Cattle shelter
	Ain Lahdjar Forest (2)		35°57'08.1"N 5°32'13.4 "E	934	Coniferous forest No human presence
Baida Bordj (L)	Baida Bordj (1)	Rural	35°53'44.7 "N 5°42'09.9 "E	862	Cattle shelter
	Zraya (2)		35°48'30.1"N 5°40'58.1"E	864	Poultry shelter

^a^ Otherwise stated, humans were present in all sites.

### Collection and identification of sand flies

**Fig 2:**
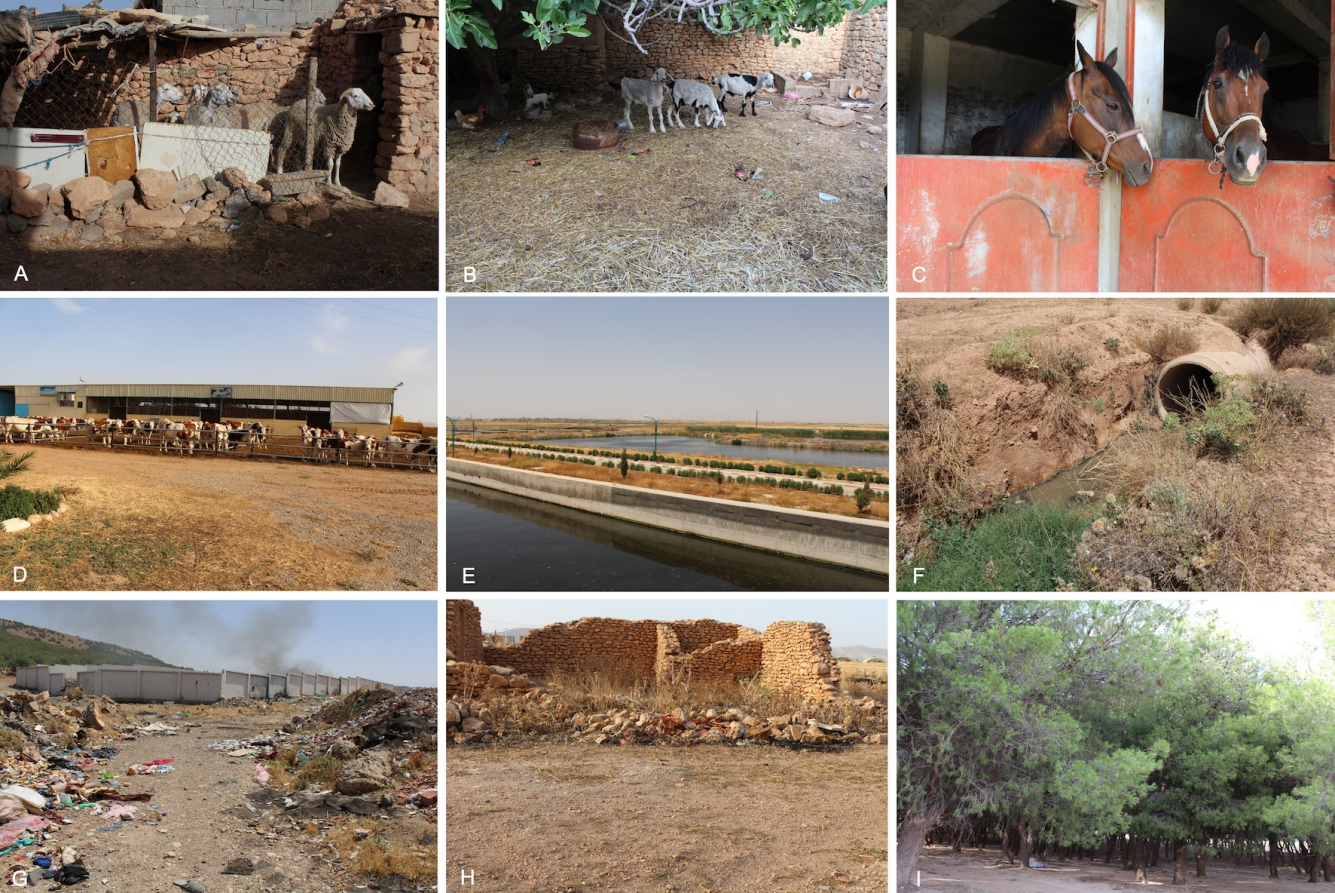
**Phlebotomine sand fly collection sites (A–I) in Setif province, North-Eastern Algeria.** (A) sheep shelter, (B) goat shelter, (C) horse shelter, (D) goat shelter, (E) water treatment station, (F) discharge of waste water, (G) discharge of domestic waste, (H) old building, and (I) coniferous forest.

Sand flies were collected from March to November 2016 and 2017, in 12 municipalities of Setif (Table 1, Fig 1). Twenty collection sites were selected, including animal stables (n = 17), an old building (n = 1), a forest (n = 1), a discharge of domestic waste (n = 1), a discharge of water waste (n = 1), and a water treatment station (n = 1) (Table 1, Fig 2). Sand flies were collected using 651 sticky paper (15 cm × 27 cm, covering up to 52.76 m^2^) and CDC lights traps as described [13,14]. CDC light traps were installed monthly, for 41 nights during the two collection seasons from 7:30 pm to 5:00 am. Collections were carried out once a month using sticky paper traps until the appearance of the first sand fly and then every 5 ± 2 days until their total disappearance. All specimens were preserved in labelled glass vials containing 70% ethanol and subsequently processed for morphological identification, using taxonomic keys and descriptions [15–18].

### Molecular detection of *Leishmania* spp. in female sand flies

Genomic DNA was extracted from thorax and abdomen (heads and last segments were removed for morphological identification) of female sand flies (n = 804) using a commercial kit (DNeasy Blood & Tissue Kit, Qiagen GmbH, Hilden, Germany), according to the manufacturer’s instructions. Detection of *Leishmania* spp. DNA was performed by conventional PCR using primers LGITSF2 (5’-GCATGCCATATTCTCAGTGTC-3’) and LGITSR2 (5’-GGCCAACGCGAAGTTGAATTC-3’) targeting partial region of the rRNA internal transcribed spacer 2 (ITS2, from 372 to 450 bp) [20]. Each PCR reaction consisted of 4 μl of sand fly genomic DNA and 46 μl of PCR mix containing 2.5 mM MgCl2, 10 mM Tris-HCl (pH 8.3), and 50 mM KCl, 250 μM of each dNTP, 50 pmol of each primer and 1.25 U of AmpliTaq Gold (Applied Biosystems, Foster City, CA, USA). PCR thermal conditions were 95°C for 10 min, followed by 35 cycles of 95°C for 30 sec, 64°C for 30 sec and 72°C for 1 min, and a final extension at 72°C for 7 min. Approximately 100 ng of sand fly genomic DNA (with the exception of the no-template control) were added to each PCR. DNA from cultured *L*. *infantum*, originally retrieved in a dog living in Italy (zymodeme MON-1), *L*. *tropica* (MHOM/IL/2005/LRC-L1239) and *L*. *major* (MHOM/TM/1973/5ASKH) promastigotes were used as positive controls. Amplified products were examined on 2% agarose gels stained with GelRed (VWR International PBI, Milan, Italy) and visualized on a GelLogic 100 gel documentation system (Kodak, New York, USA). Amplicons were purified and sequenced in both directions using the same primers as for PCR, employing the Big Dye Terminator v.3.1 chemistry in an automated sequencer (3130 Genetic Analyzer, Applied Biosystems, Foster City, CA, USA). The ITS2 *Leishmania* sequences were aligned using the ClustalW program [21] and compared with those available in GenBank using the BLASTn tool (http://blast.ncbi.nlm.nih.gov/Blast.cgi).

The phylogenetic relationships of *Leishmania* spp. were inferred by Maximum Likelihood (ML) method based on the Tamura 3-parameter model [22] with Invariant sites (I), selected by best-fit model [23]. Evolutionary analysis was tested with 8000 bootstrap replications, using MEGA6 software [24]. The phylogenetic analysis was run using ITS2 reference sequences of *Leishamania* species available in GenBank. The sequence from *Trypanosoma evansi* (Accession number LC199491) was used as outgroup.

### Human cases

To assess the relationship occurring between the diversity of vector populations and the risk of human infection, CL cases notified in Setif province were included in the study. From 2016 to 2017, data regarding patients (i.e., sex, age and place of residence) were obtained from the Direction of Health and Population (DHP) of Setif. Data were obtained and processed anonymously, with the authorization of the head of the prevention department from the same direction (Ref. 1173/HPD/PD/2019).

### Meteorological data

**Fig 3:**
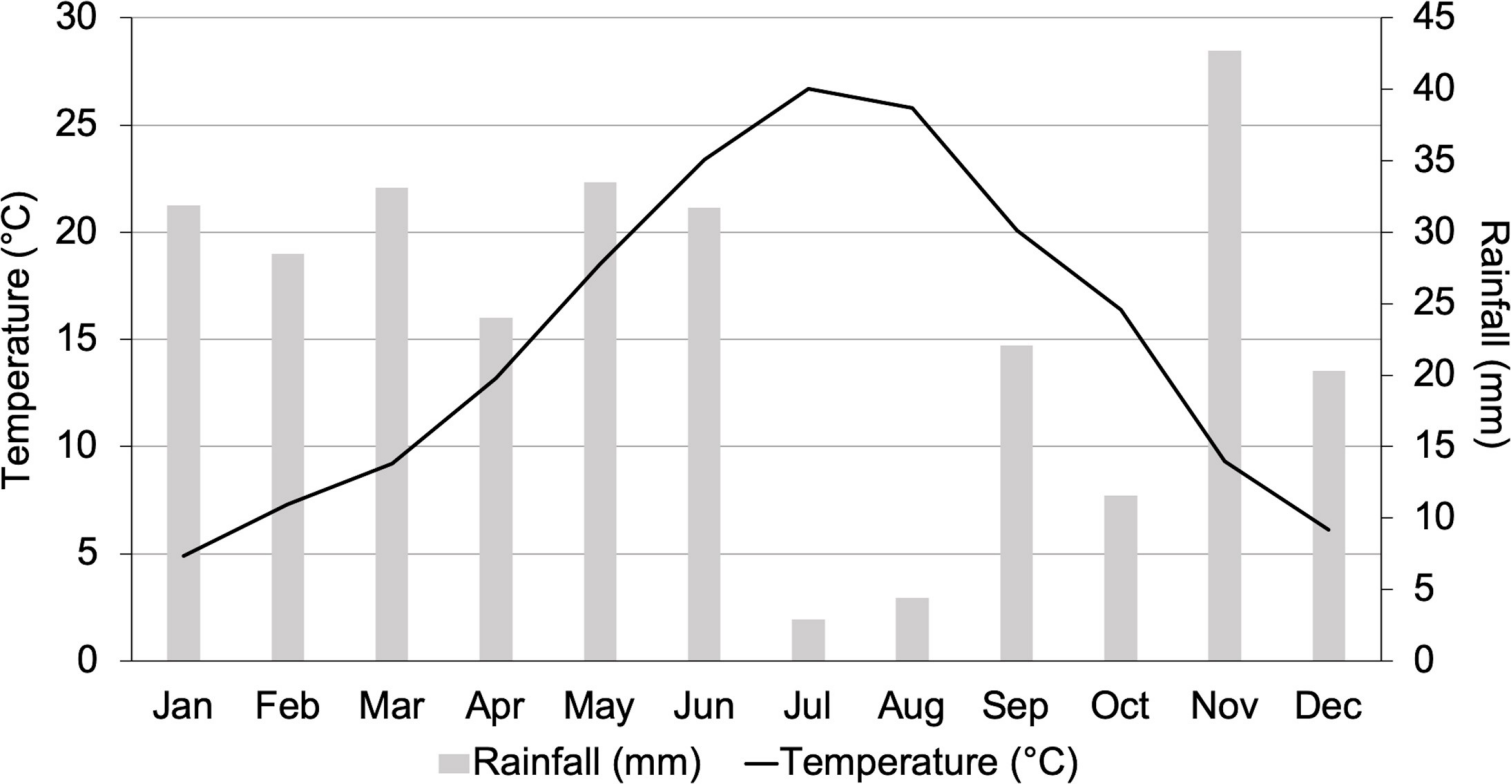
Monthly mean temperature and relative humidity in Setif province, North-Eastern Algeria, based on average data recorded during 2016 and 2017.

From March to November 2016 and 2017, temperature (C°), relative humidity (%), rainfall (mm) and wind speed (m/s) data were obtained from National Office of Meteorology (ONM) of Setif. These data are firstly registered in the auxiliary station located in Ain Oulmen (site G), then reported to the central station situated in the Setif International Airport (World Meteorological Organization code: 60445). Temperature, relative humidity and wind speed were registered daily every 3 hours, whilst precipitation data were recorded twice per day. The ombrothermic diagram [25] has been established according to monthly mean temperature and relative humidity, based on average data recorded during 2016 and 2017 (Fig 3).

### Data analysis

The spatial distribution of sand flies was estimated, according to the species, by the pattern of occurrence (C %) expressed by the formula C = n/N (i.e., n = number of sites positive for sand flies of each species; N = total number of positive sites) as sporadic (C = 0–20%), infrequent (C = 20.1–40%), moderate (C = 40.1–60%), frequent (C = 60.1–80%) or constant (C = 80.1–100%) [26].

Sand fly species dominance was estimated by the relative abundance (RA %), expressed by the ratio between number of specimens for each species and the total number of specimens caught ×100 [27]. Sex ratio was calculated by dividing the number of females by the number of males. Lilliefors test was used to test the normality of the data. The total number of specimens for each species collected monthly and the number of females and males according to species were compared using non-parametric tests (Kruskal-Wallis H-test, Mann-Whitney U-test) respectively. Spearman’s (*rs*) correlation coefficients were used to assess the relationship between the number of sand flies and meteorological variables (i.e., average monthly temperature, relative humidity, rainfall and wind speed). Statistical analyses were performed using IBM SPSS statistics 23^rd^ version for Windows 10. Differences were considered statistically significant when *P*<0.05.

## Results

Out of 1804 sand flies collected, 1737 (96.2%) were morphologically identified as belonging to seven species. Among these, the most abundant species were *Ph*. *perniciosus* (76.2%), followed by *Ph*. *papatasi* (16.7%) and *Ph*. *sergenti* (5%), representing together 97.9% of the collected specimens (Table 2). The remaining specimens were identified as *Ph*. *longicuspis*, *Ph*. *perfiliewi*, *Se*. *minuta* and *Se*. *fallax*. *Phlebotomus perniciosus* was present with a constant pattern of occurrence (C = 87.5%) throughout the study period, whereas a moderate occurrence was recorded for *Ph*. *papatasi* and *Ph*. *sergenti* (C = 42.9%) and an infrequent occurrence for *Se*. *minuta* (C = 28.6%). *Phlebotomus longicuspis*, *Ph*. *perfiliewi* and *Se*. *fallax* occurred sporadically (C = 7.1%) (Table 2).

**Table 2:** Phlebotomine sand flies collected in Setif province, North-Eastern Algeria, 2016–2017, according to species, sex, pattern of occurrence (C) and relative abundance (RA).

Species	*n*	M	F	C (%)	Total RA (%)	RA 2016 (%)				RA 2017 (%)	
						A	B	C	E	A	B
*Ph*. *perniciosus*	1324	600	724	87.5	73.4	95.0	4.1	0.2	-	71.7	28.3
*Ph*. *papatasi*	290	245	45	42.9	16. 7	95.9	4.5	-	-	98.1	1.9
*Ph*. *sergenti*	87	59	28	42.9	4.8	80	20	-	-	52.2	47.8
*Ph*. *longicuspis*	4	1	3	7.1	0.2	66.7	33.3	-	-	-	-
*Ph*. *perfiliewi*	2	1	1	7.1	0.1	-	-	-	-	100	-
*Se*. *minuta*	23	9	14	28.6	1.3	62.5	37.5	-	-	40	60
*Se*. *fallax*	7	1	6	7.1	0.4	100	-	-	-	100	-
*Phlebotomus* spp.	62	-	-	35.7	3.4	90	10	-	-	63.3	36.7
*Sergentomyia* spp.	5	-	-	35.7	0.3	33.3	33.3	-	33.3	-	-

C, pattern of occurrence; RA, relative abundance; -, not calculated, *Ph*., *Phlebotomus*; *Se*., *Sergentomyia*.

Out of 20 collection sites, eight (i.e., A1, A2, A3, B1, B2, C1, C2, E) and three (i.e., A1, A2, B1) sites located in rural areas were positive for sand flies in 2016 and 2017, respectively (Table 2). No urban and peri-urban area scored positive for sand flies during the study period. All sand fly-positive sites were located in mountainous areas in the extreme south of Setif, neighbouring north Saharan regions. The highest number of taxa and RA of specimens were registered in sites with animal shelters for the first year of collection, with a slight increasing of RA in second year for *Ph*. *papatasi* (98.1%) and *Ph*. *perfiliewi* (100%) from site A and for *Ph*. *perniciosus* (28.28%), *Ph*. *sergenti* (47.8%) and *Se*. *minuta* (60%) from site B.

**Fig 4:**
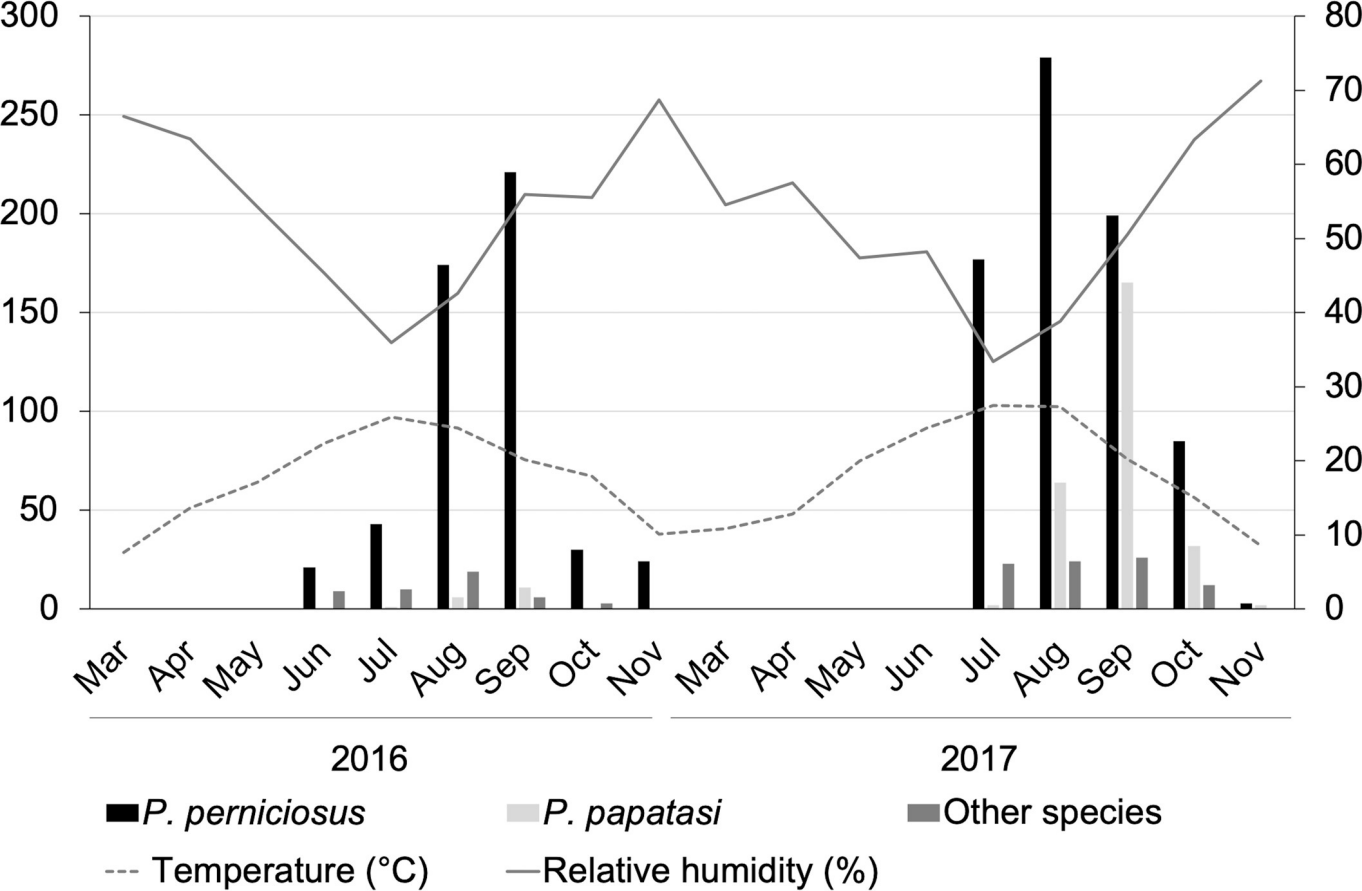
Monthly dynamics of sand flies (*Phlebotomus perniciosus*, *Phlebotomus papatasi*, and remaining species combined) in Setif province, North-Eastern Algeria, 2016–2017, in relation to temperature and relative humidity.

The number of specimens for each species collected monthly vary significantly in 2016 (Kruskal-Wallis H-test, *H* = 13.78, *df* = 6, *P* = 0.03) or in 2017 (Kruskal-Wallis H-test, *H* = 13.24, *df* = 6, *P* = 0.03). Sand flies were present during the summertime period (April–October), but not during the winter period (November–March) (Fig 4). In particular, sand flies were collected from the middle of June and until the first days of November in 2016, peaking in August, when average monthly temperature and humidity of 24.4°C and 42.6% were registered. In 2017, sand flies occurred from the middle of July to the end of November with two peaks in August (average monthly temperature and relative humidity of 27.3°C and 38.8%, respectively) and in September (average monthly temperature and relative humidity 20.2°C and 50.5%, respectively). Number of sand flies collected in 2016 and 2017, mean daily temperature and relative humidity recorded at each day of collection are reported in supplementary tables (S1 and S2 Tables).

The monthly number of sand flies (all species combined) was positively correlated with temperature (*rs*_(16)_ = 0.541, *P*<0.05). The same trend was also observed when data from *Ph*. *perniciosus* (i.e., the most abundant species) was analysed (*rs*_(16)_ = 0.556, *P* <0.05 for temperature). No significant correlations were found between the monthly number of sand flies (all species combined) and rainfall (*rs*_(16)_ = −0.286, *P* = 0.25), relative humidity (*rs*_(16)_ = −0.376, *P* = 0.13) or wind speed (*rs*_(16)_ = −0.169, *P* = 0.50).

The number of females was lower than males with an overall female:male ratio of 0.89, with the exclusion of *Ph*. *longicuspis*, *Se*. *minuta*, *Se*. *fallax*, for which a higher number of females specimens were collected (Table 2). The number of females and males did not vary significantly according to species (Mann-Whitney U-test, *U* = 22.5, *n*_1_ = 7, *n*_2_ = 7, *P* = 0.80).

Out of 801 females tested, nine *Ph*. *perniciosus* (1.1%) collected from two municipalities (A1, A2) scored positive for *Leishmania* spp. DNA. In particular, five specimens were positive for *L*. *infantum*, three for *L*. *major* and one for *L*. *tropica* (Table 3).

**Table 3:** Phlebotomine sand flies positive for *Leishmania* spp., accordingly to year and site of collection in Setif, North-Eastern Algeria. Accession numbers of ITS2 reference sequences and percentage of identity are reported.

Specimen ID	Sand fly species	E/NE	*Leishmania* species	Accession number/identity (%)	Collection site
**2016**					
69	*Ph*. *perniciosus*	NE	*L*. *major*	FR796423.1 (99.1)	Lahddada, Boutaleb
73	*Ph*. *perniciosus*	NE	*L*. *major*	FR796423.1 (98.4)	Lahddada, Boutaleb
78	*Ph*. *perniciosus*	E	*L*. *infantum*	MK645051.1 (99.7)	Lahddada, Boutaleb
117	*Ph*. *perniciosus*	NE	*L*. *major*	FR796423.1 (100)	Lahddada, Boutaleb
134	*Ph*. *perniciosus*	NE	*L*. *tropica*	FJ948452.1 (99.4)	Lahddada, Boutaleb
159	*Ph*. *perniciosus*	NE	*L*. *infantum*	MH605316.1 (99.7)	Lahddada, Boutaleb
**2017**					
277	*Ph*. *perniciosus*	NE	*L*. *infantum*	MK481044.1 (100)	Bni Lmai, Boutaleb
299	*Ph*. *perniciosus*	E	*L*. *infantum*	MK481044.1 (100)	Lahddada, Boutaleb
325	*Ph*. *perniciosus*	E	*L*. *infantum*	MK481044.1 (100)	Lahddada, Boutaleb

E, engorged; NE, non engorged.

**Fig 5:**
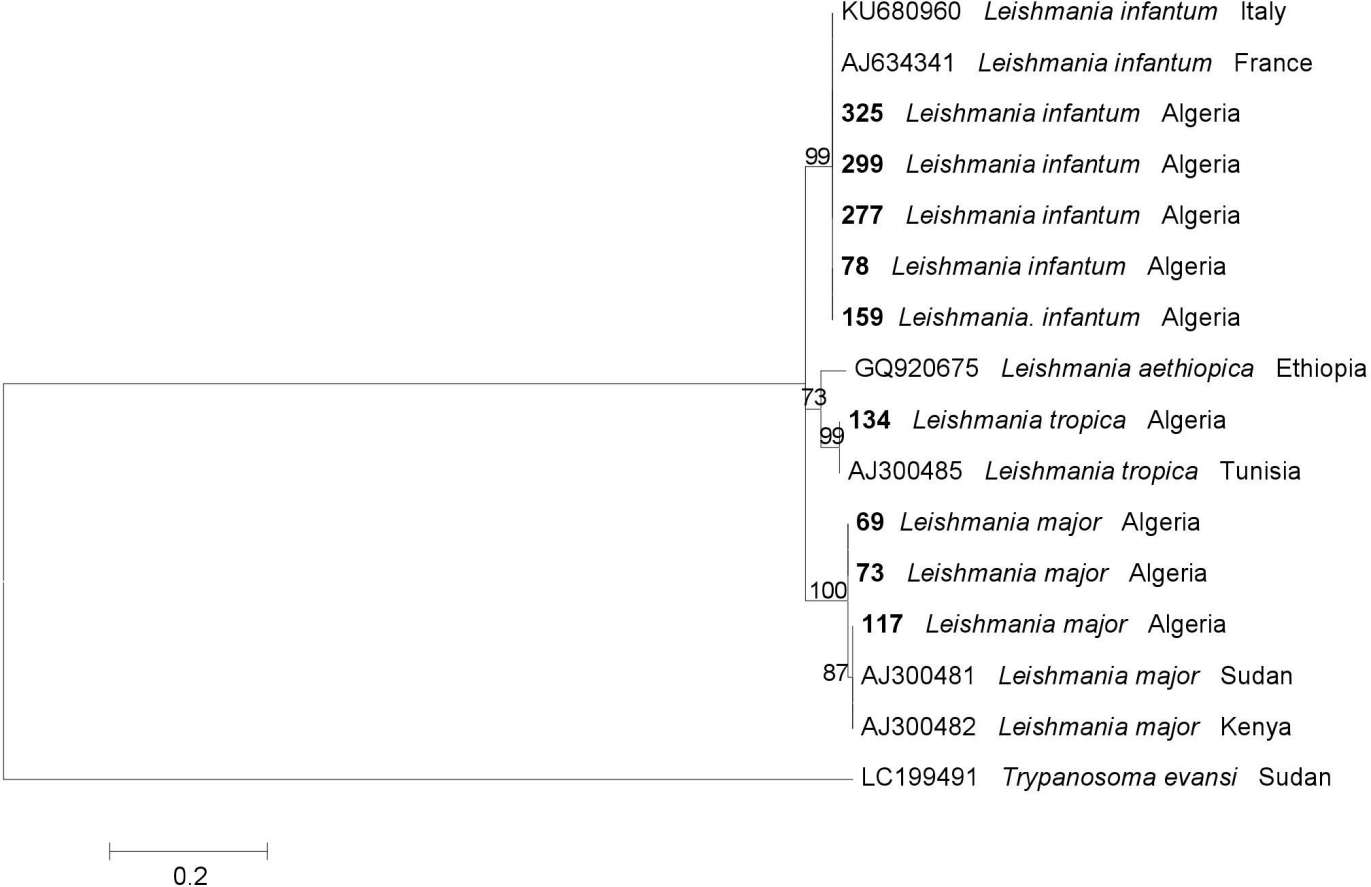
Maximum Likelihood tree based on ITS2 sequences of *Leishmania* spp. generated herein and sequences from GenBank (accession numbers provided). Bootstrap values are based on 8000 replicates and only bootstraps >50% are indicated. *Trypanosoma evansi* was used as outgroup. Identification numbers of sand flies positive for *Leishmania* spp. are reported in bold.

Reflecting the genetic identification, the phylogram of ITS2 sequences grouped each *Leishmania* species examined in three paraphyletic clades including those of each corresponding reference *Leishmania* spp. (Fig 5).

During the study period, 34 CL human cases were notified in nine of 12 municipalities investigated in this study (Table 4). Of these, 14 (41.2%), 15 (44.1%) and five (14.7%) CL cases were registered in five rural areas (i.e., sites A–E) and in two urban (i.e., sites F, G) and peri-urban (i.e., sites H, I) municipalities, respectively (Table 4). In 2016, seven CL cases (20.6%) occurred in March (n = 1), in November (n = 3) and in December (n = 3). These cases were notified for males from 10 to 50 years old living in rural (71.4%, n = 5) and urban (28.6%, n = 2) areas (Table 4). In 2017, 27 CL cases (79.4%) occurred from October to December (n = 13) and from January to March (n = 14). CL cases were notified both for males (66.7%, n = 18) and for females (33.7%, n = 9). These human cases are distributed among all age groups of patients examined living in rural (33.3%, n = 9), urban (48.1%, n = 13) and peri-urban (18.5%, n = 5) areas.

**Table 4:** Human cutaneous leishmaniasis cases notified in Setif province, North-Eastern Algeria, 2016–2017, accordingly to municipality, month of notification, sex and age.

Municipalities	Site type	2016						2017						
		Human cases		Age (years) and sex (M, F)				Human cases		Age (years) and sex (M, F)				Total
		Month	*n*	<10	10–30	30–50	50–70	Month	*n*	<10	10–30	30–50	50–70	
Boutaleb	Rural	-	-	-	-	-	-	Jan	1	-	-	-	1F	1
Hamma	Rural	Nov	1	-	-	1M	-	Oct	2	1F	1M	-	-	3
Ouled Tebbane	Rural	Nov	1	-	1M	-	-	Jan	3	-	1F	2F	-	4
Rasfa	Rural	Dec	2	-	2M	-	-	Jan-Dec	3	1M	1M	-	1F	5
Ouled Sid Ahmed	Rural	Mar	1	-	-	1M	-	-	-	-	-	-	-	1
Saleh Bey	Urban	Nov	1	1M	-	-	-	Nov	1	-	-	1M	-	2
Ain Oulmen	Urban	Dec	1	-	1M	-	-	Jan-Mar, Nov-Dec	12	1F	4M, 1F	5M, 1F	-	13
Kser El Abtal	Periurban	-	-	-	-	-	-	Oct-Dec	4	2M	1M	1M	-	4
Bir Haddada	Periurban	-	-	-	-	-	-	Jan	1	-	1M	-	-	1
**Total**			7	1	4	2	-		27	5	10	10	2	34

-, not available; M, male; F, female.

## Discussion

A high degree of sand fly species richness was recorded in Setif, with a higher relative abundance registered in rural areas of two municipalities (i.e., Boutaleb and Hamma), where sheep, goat and horses were the most common animals found. Even if no significant variations in terms of meteorological variables (i.e., temperature, relative humidity, rainfall and wind speed) were recorded between 2016 and 2017 in Setif, the spatio-temporal dynamics and the abundance of sand flies changed between the both seasons. Indeed, a decrease in number of positive collection sites and of sand fly species and abundance was recorded during the second year. This may be related to the relatively low abundance of sand flies collected during the whole study period. On the other hand, the absence of sand flies recorded for urban and peri-urban municipalities could also be due to the chemical treatment (deltamethrin) applied in these sites (i.e., A2, A3, B2 and C) during May and June 2017. However further studies, perhaps using local data loggers, would be valuable to confirm these analyses, which were done using data from meteorological stations.

Overall, of 24 sand fly species present in Algeria [15,28], seven (i.e., *Ph*. *perniciosus*, *Ph*. *papatasi*, *Ph*. *sergenti*, *Ph*. *longicuspis*, *Ph*. *perfiliewi*, *Se*. *minuta* and *Se*. *fallax*) were identified in the present study, with most of them being found in sympatry in different municipalities. A similar sand fly population structure has been observed also in the northern/north-eastern and middle-eastern regions of Algeria, where up to ten sand fly species were identified (i.e., Biskra region) [27,29,30].

The occurrence of *Ph*. *perniciosus* as the most abundant species may be partly explained by the capacity of this sand fly to adapt to different environments such as stables and human houses. In addition, the high abundance of *Ph*. *perniciosus* was probably due to its endo-exophilic and anthropozoophilic behaviour [11,27,31] and because it is well adapted to the zones having from sub-humid to semi-arid climate [15,29]. A similar abundance (up to 77%) for this sand fly species was also recorded in other provinces in Northern and North-Eastern Algeria [11,28,31]. *Phlebotomus papatasi* was the second most common species collected. This finding is in contrast with those obtained from other studies carried out in North and North-Eastern Algeria where frequencies of 0.2% and 1.2% for *Ph*. *papatasi* were registered in Constantine, Tipaza and Tizi-Ouzou provinces, respectively [11,31,32]. The abundance of *Ph*. *papatasi* in Setif may be explain by its ability to adapt to semi-arid climate. Indeed, it is well known that *Ph*. *papatasi*, an anthropophilic and endophilic species, is typical of Saharan and arid areas and rarely collected in semi-arid regions [8,15,31].

The finding of *Ph*. *sergenti* with a frequency of 5% is in line with data from previous studies conducted in north and north-east regions of Algeria, where a relative frequency of 4.8% was registered [8,29,32,33]. The relatively low frequency of this species in Setif may be due to the semi-arid climate of the region, as *Ph*. *sergenti* is a species which typically occurs in Saharan and arid zones and lives in wall crevices, rodent burrows and rocky areas [15]. On the other hand, the low numbers of *Ph*. *longicuspis* and *Ph*. *perfiliewi*, which occurred during the whole study period, may be due to the unsuitable environmental conditions and/or host presence [15,28,29].

The finding of *Se*. *minuta* and *Se*. *fallax* with relative abundances of up 62.5% and 100%, respectively (see Table 2), agrees with previous data revealing that *Sergentomyia* species are abundant in all the Algerian territory and have adapted in all the bioclimatic areas, from semi-arid and Saharan to sub-humid and humid bioclimatic stages [27,34,35].

Considering that the presence of a competent vector is a determinant factor for the local circulation of a given vector-borne pathogen, the retrieval of proven vectors in different areas of Setif may explain the local circulation in this province of different *Leishmania* spp., whose genetic identification was supported by the phylogenetic analysis. Furthermore, the presence of *Ph*. *sergenti* and *Ph*. *papatasi*, the proven vectors of *L*. *tropica* and *L*. *major* [36,37], suggest the circulation of these parasites in this region. This was reinforced by the detection of *L*. *tropica* and *L*. *major* DNA in *Ph*. *perniciosus*. Noteworthy, the mere detection of *L*. *tropica* and *L*. *major* DNA does not imply that *Ph*. *perniciosus* is a vector. The detection of different *Leishmania* spp. in non-competent vectors has been previously reported [38–40].

Even if no sand flies were collected in urban and peri-urban areas of Setif, CL cases have been registered in these areas. However, the possibility that these CL patients may have acquired the infection in rural environments cannot be ruled out. Indeed, most of the 34 cases of CL herein analysed were young males between 10 and 30-years old, employed in agricultural activities, especially during the summer, and consequently exposed at high risk of sand fly bites.

The high number of CL cases registered in Algeria may be explained by the extension of classical foci and by the emergence of new foci across the country [26,41,42]. The occurrence of different bioclimatic zones (e.g., Mediterranean climate in the north and humid and semi-arid zones in the southern Sahara), the desertification in the steppe of the northern Sahara, and population movements from endemic to non-endemic areas (and *vice versa*) have likely contributed to the increase in the number of CL cases in Algeria in the past decades [6,29,32,33,43,44].

This study confirms the presence of different proven vectors of *Leishmania* spp. (e.g., *Ph*. *perniciosus*, *Ph*. *papatasi* and *Ph*. *sergenti*) in several municipalities of Setif, highlighting the risk of infection in humans and animals by three different *Leishmania* species (i.e., *L*. *infantum*, *L*. *major* and *L*. *tropica*). Sand flies were positively correlated with temperature and public health authorities should benefit from this information to establish optimized vector control strategies in Setif and surrounding risk areas in Algeria.

## Supporting information

S1 Table Density, number, sex ratio of phlebotomine sand flies collected in 2016. **Mean daily of temperature and relative humidity recorded at each day of collection were reported.** Ph, phlebotomine sand flies.
(DOCX)

S2 Table Density, number, sex ratio of phlebotomine sand flies collected in 2017. **Mean daily of temperature and relative humidity recorded at each day of collection were reported.** Ph, phlebotomine sand flies.
(DOC)
