# Supplementary material for: Phlebotomine sand flies and Leishmania species in a focus of cutaneous leishmaniasis in Algeria
Source: PLoS Negl Trop Dis. 2020 Feb 18;14(2):e0008024. doi: 10.1371/journal.pntd.0008024 (PMC7048314; doi:10.1371/journal.pntd.0008024)
Supplement: S1 Table — Mean daily of temperature and relative humidity recorded at each day of collection were reported. Ph, phlebotomine sand flies. (DOCX) [file pntd.0008024.s001.docx]

**S1 Table**

| **Date** | **Density**  **(Ph/m^2^)** | **Sand fly number** | **M** | **F** | **Sex ratio** | **Temperature**  **(°C)** | **Precipitation**  **(mm)** | **Humidity (%)** | **Wind speed**  **(m/s)** |
| --- | --- | --- | --- | --- | --- | --- | --- | --- | --- |
| 13-06-2016 | 16.46 | 2 | 0 | 2 | - | 23.4 | 0 | 40 | 3.8 |
| 16-06-2016 | 12.34 | 3 | 1 | 2 | 2 | 21.2 | 0 | 51 | 3.5 |
| 20-06-2016 | 49.38 | 8 | 6 | 2 | 0.33 | 15.2 | 0 | 59 | 3.8 |
| 23-06-2016 | 39.50 | 8 | 2 | 6 | 3 | 22.5 | 0 | 41 | 3.1 |
| 27-06-2016 | 10.97 | 7 | 4 | 3 | 0.75 | 24.6 | 0 | 47 | 2.7 |
| 10-07-2016 | 9.87 | 2 | 1 | 1 | 1 | 29 | 0 | 32 | 4.2 |
| 13-07-2016 | 11.52 | 17 | 2 | 15 | 7.5 | 25.2 | 0 | 42 | 3.6 |
| 14-07-2016 | 34.56 | 5 | 2 | 3 | 1.5 | 20.8 | 0 | 59 | 3.8 |
| 17-07-2016 | 19.75 | 4 | 0 | 4 | - | 24.2 | 0 | 41 | 3.3 |
| 20-07-2016 | 7.57 | 27 | 14 | 13 | 0.92 | 26 | 0 | 30 | 2.7 |
| 24-07-2016 | 29.62 | 6 | 6 | 0 | 0 | 22.4 | 0 | 55 | 3.7 |
| 27-07-2016 | 15.28 | 13 | 7 | 6 | 0.85 | 24.2 | 0 | 39 | 2.6 |
| 29-07-2016 | 6.17 | 7 | 2 | 3 | 1.5 | 28.3 | 0 | 30 | 3.5 |
| 04-08-2016 | 62.61 | 71 | 23 | 48 | 2.08 | 26.8 | 0 | 27 | 2.6 |
| 12-08-2016 | 24.69 | 11 | 0 | 11 | - | 20.5 | 0 | 51 | 2.5 |
| 17-08-2016 | 66.21 | 59 | 11 | 48 | 4.36 | 24.4 | 0 | 41 | 4.1 |
| 19-08-2016 | 10.80 | 7 | 0 | 7 | - | 27.3 | 0 | 38 | 3.3 |
| 21-08-2016 | 24.69 | 27 | 7 | 20 | 2.85 | 25.8 | 0 | 38 | 2.8 |
| 26-08-2016 | 52.46 | 34 | 9 | 25 | 2.77 | 25.1 | 0 | 40 | 3.7 |
| 01-09-2016 | 3.52 | 1 | 0 | 1 | - | 24 | 0 | 51 | 3 |
| 10-09-2016 | 153.69 | 61 | 44 | 17 | 0.38 | 23.3 | 0 | 43 | 3.1 |
| 11-09-2016 | 148.14 | 42 | 33 | 9 | 0.27 | 21.7 | 0 | 54 | 3.2 |
| 15-09-2016 | 38.8 | 22 | 5 | 17 | 3.5 | 21.9 | 0 | 64 | 4.1 |
| 22-09-2016 | 2.93 | 2 | 2 | 0 | 0 | 20.5 | 22 | 40 | 2.7 |
| 24-09-2016 | 10.58 | 3 | 2 | 1 | 0.5 | 15.9 | 74 | 78 | 3 |
| 27-09-2016 | 22.04 | 9 | 7 | 2 | 0.28 | 16.3 | 0 | 67 | 3.3 |
| 28-10-2016 | 22.04 | 5 | 2 | 3 | 1.5 | 13.4 | 0 | 64 | 4.2 |
| 01-11-2016 | 2.44 | 9 | 7 | 2 | 0.28 | 14.6 | 0 | 53 | 2.1 |
