# Supplementary material for: Phlebotomine sand flies and Leishmania species in a focus of cutaneous leishmaniasis in Algeria
Source: PLoS Negl Trop Dis. 2020 Feb 18;14(2):e0008024. doi: 10.1371/journal.pntd.0008024 (PMC7048314; doi:10.1371/journal.pntd.0008024)
Supplement: S2 Table — Mean daily of temperature and relative humidity recorded at each day of collection were reported. Ph, phlebotomine sand flies. (DOC) [file pntd.0008024.s002.doc]

| **Date** | **Density**  **(*Ph*/m^2^)** | **Sand fly number** | **M** | **F** | **Sex ratio** | **Temperature (°C)** | **Precipitation**  **(mm)** | **Humidity (%)** | **Wind speed (m/s)** |
| --- | --- | --- | --- | --- | --- | --- | --- | --- | --- |
| 17-07-2017 | 6.73 | 12 | 3 | 9 | 3 | 24 | 0 | 39 | 2.7 |
| 22-07-2017 | 17.81 | 13 | 10 | 3 | 0.3 | 32.5 | 0 | 21 | 5.8 |
| 24-07-2017 | 14.47 | 17 | 6 | 7 | 1.16 | 29.6 | 0 | 32 | 5 |
| 26-07-2017 | 35.08 | 27 | 17 | 10 | 0.58 | 23.4 | 0 | 52 | 3.2 |
| 29-07-2017 | 18.34 | 26 | 19 | 7 | 0.36 | 29.7 | 0 | 28 | 3.6 |
| 31-07-2017 | 66.81 | 46 | 28 | 18 | 0.64 | 31.9 | 3 | 23 | 4.3 |
| 05-08-2017 | 39.16 | 46 | 29 | 17 | 0.58 | 30.4 | 0 | 28 | 3 |
| 08-08-2017 | 19.20 | 21 | 14 | 7 | 0.5 | 29 | 1 | 30 | 4.1 |
| 11-08-2017 | 5.92 | 6 | 1 | 5 | 5 | 23.7 | 0 | 62 | 3.7 |
| 16-08-2017 | 13.16 | 8 | 4 | 4 | 1 | 30.1 | 0 | 33 | 3.1 |
| 23-08-2017 | 3.70 | 3 | 0 | 3 | - | 25.1 | 0 | 39 | 3.1 |
| 25-08-2017 | - | - | - | - | - | 27.4 | 0 | 34 | 2.7 |
| 26-08-2017 | 20.33 | 14 | 5 | 9 | - | 25.4 | 2 | 47 | 3.6 |
| 31-08-2017 | 115.22 | 42 | 42 | 0 | 0 | 25.1 | 0 | 38 | 2.7 |
| 03-09-2017 | 30.86 | 14 | 9 | 5 | 1.8 | 27 | 0 | 36 | 2.7 |
| 06-09-2017 | 69.19 | 51 | 37 | 14 | 0.37 | 25.1 | 0 | 38 | 2.7 |
| 10-09-2017 | 79.36 | 54 | 40 | 14 | 0.35 | 20.4 | 0 | 52 | 2.8 |
| 14-09-2017 | 158.73 | 117 | 69 | 48 | 0.69 | 23 | 9 | 47 | 2.3 |
| 18-09-2017 | 38.48 | 24 | 15 | 9 | 0.6 | 14.3 | 35 | 69 | 5 |
| 23-09-2017 | 72.01 | 49 | 42 | 7 | 0.16 | 22.5 | 0 | 46 | 3.2 |
| 02-10-2017 | 28.88 | 23 | 20 | 3 | 0.15 | 20.1 | 0 | 61 | 3.6 |
| 07-10-2017 | 47.13 | 42 | 28 | 14 | 0.5 | 23 | 254 | 39 | 4.1 |
| 13-10-2017 | 13.60 | 19 | 13 | 6 | 0.46 | 14.9 | 9 | 75 | 2.2 |
| 18-10-2017 | 44.09 | 15 | 13 | 2 | 0.15 | 15 | 0 | 68 | 2.3 |
| 23-10-2017 | 9.66 | 9 | 8 | 1 | 0.12 | 13.9 | 78 | 52 | 3 |
| 28-10-2017 | 7.51 | 7 | 5 | 2 | 0.4 | 12.9 | 61 | 41 | 4.2 |
| 02-11-2017 | 17.36 | 2 | 1 | 1 | 1 | 13.5 | 70 | 39 | 3.1 |
| 07-11-2017 | 1.07 | 1 | 0 | 1 | - | 5.7 | 63 | 71 | 2.7 |
| 12-11-2017 | 0.93 | 1 | 1 | 0 | 0 | 10.1 | 82 | 64 | 3.5 |

**S2 Table**
